# Philip Morris involvement in the development of an air quality laboratory in El Salvador

**DOI:** 10.1136/tc.2008.026989

**Published:** 2009-02-11

**Authors:** C E Kummerfeldt, J Barnoya, L Bero

**Affiliations:** 1 Cardiovascular Unit of Guatemala, Guatemala City, Guatemala; 2 University of California, San Francisco, Center for Tobacco Control Research and Education and Department of Epidemiology, San Francisco, California, USA; 3 University of California, San Francisco, California, USA

## Abstract

**Background:**

The tobacco industry has organised research institutions to generate misleading data on indoor air quality, including second-hand smoke exposure and health effects.

**Objectives:**

To describe tobacco industry involvement in the organisation and financial support of an air quality research laboratory in El Salvador.

**Methods:**

Tobacco industry documents on the internet were systematically searched from August 2007 to February 2008 for air quality studies undertaken in El Salvador, and laboratory personnel were interviewed.

**Results:**

Philip Morris sought to establish a network of air quality laboratories throughout Latin America. In El Salvador, in 1997, through Tabacalera de El Salvador (a subsidiary of Philip Morris) and the Salvadoran Foundation for Economic Development (FUSADES), the industry organised an air quality research laboratory. FUSADES was part of the industry’s Latin American Scientific Network, which consisted of doctors hired as consultants who would send air samples from their research to FUSADES. Philip Morris Scientific Affairs personnel hired LabStat, a Canadian-based laboratory, to provide technical assistance to FUSADES (train and assist the laboratory in air quality measurements). In addition, the Washington-based HMS Group successfully implemented a plan to upgrade the laboratory and obtain international certifications. HMS Group also assisted in searching for sustainable funding for FUSADES, including seeking funds from international aid for Hurricane Mitch.

**Conclusion:**

Air quality studies that have used the FUSADES laboratory should be carefully interpreted, given the support that this laboratory received from Philip Morris.

The tobacco industry has created its own research centres and set its own quality standards to fuel controversy on the health effects of tobacco.^1^ In 1988, the industry created the Center for Indoor Air Research (CIAR) which funded projects to deflect attention from second-hand smoke (SHS) as an indoor air pollutant.^2^ Similar efforts by the industry include assistance in the International Society of the Built Environment and the Australian Tobacco Research Foundation.^3^ ^4^ The industry has also influenced the American Society of Heating, Refrigeration, and Air Conditioning Engineers (ASHRAE) in the development of ventilation standards for indoor air quality in the USA.^5^

In Latin America the tobacco industry has led research efforts to obstruct implementation of smoke-free environments. In the 1990s, British American Tobacco (BAT) and Philip Morris (PM) hired doctors and scientists to undermine efforts to regulate SHS in the region.^6^ This same strategy has been used worldwide to generate evidence that supports the industry’s position that SHS is a minor contributor to indoor air pollution and focus the debate on outdoor air pollution.^7^ ^8^

In 1997 PM became interested in establishing a network of air quality measurement laboratories to conduct indoor and outdoor air research in Latin America.^9^ Therefore, until 1999 PM supported the El Salvador-based FUSADES (Foundation for Economic and Social Development) laboratory in order to turn it into a regional centre for air quality testing.^10^ In this paper, we describe PM’s involvement in converting this laboratory into its first Latin American certified air quality measurement centre.

## METHODS

Between August 2007 and February 2008 we searched internal tobacco industry documents available from the University of California San Francisco’s Legacy Tobacco Documents Library (http://legacy.library.ucsf.edu/), Tobacco Documents Online (http://www.tobaccodocuments.org) and the BAT Documents Repository (http://bat.library.ucsf.edu). We initially searched using the key words “FUSADES”, “Jorge Zablah” and “El Salvador”. After identifying key players and events, we used a snowball strategy to search for associated names, locations, dates and reference (Bates) numbers near relevant documents. We also used the internet search engine Google (http://www.google.com) to look for air quality studies conducted by the FUSADES laboratory.

We identified several hundred documents including correspondence, internal communications, letters, memoranda, reports, presentations, budgets and personal notes. We then narrowed our review to approximately 200 relevant documents. They were organised in chronological order and iteratively coded. Our analysis consisted of reviewing and summarising the documents, generating recurring themes from the data and grouping the documents by themes.

We interviewed via telephone two FUSADES employees who were involved during the period when PM supported the laboratory. Questions were aimed at clarifying and supplementing information from the industry documents. They were also asked to provide studies or references on indoor and outdoor air quality in which the laboratory had participated.

## RESULTS

FUSADES, established in 1983, is a non-profit organisation founded by several private enterprises in El Salvador.^11^ ^12^ According to Eduardo Nuñez Iraheta, FUSADES Executive Director in 1995, it was established “…with the aim of promoting, within a system of individual liberties, economic and social development in order to improve the living conditions for all [S]alvadorans”.^12^ The Laboratory of Integral Quality, created by FUSADES in 1990, would provide the quality control needs of the agricultural, industrial and government sectors.^11^ Services provided include soil, water, microbiology and chemical and physical analysis.^13^

SWISSCONTACT, Switzerland’s private sector organisation for development cooperation, with financial support from the Swiss Agency for Development and Cooperation, began to monitor air quality in the capital cities of Guatemala, El Salvador, Honduras, Nicaragua, Costa Rica and Panama in 1993.^14^ In El Salvador, in 1996, the laboratory monitored ozone, nitrogen dioxide, carbon monoxide, total suspended particles, PM_10_ and lead.^15^ Support from SWISSCONTACT ended in 2003 as part of a planned phase out (Jon Bickel, SWISSCONTACT Peru, personal communication).^14^ ^15^ Air quality monitoring continued until 2004 with support from the Ministry of Environment and Natural Resources.^13^ ^16^

According to the 1995 FUSADES annual report, all the organisation’s activities were financed through contributions from private sponsors, including Tabacalera de El Salvador (PM’s local subsidiary).^12^ The General Manager of Tabacalera de El Salvador was Jorge Zablah^17^ also founding member, Chair and Executive director of FUSADES from 1995–1997.^12^ In 1995, Zablah lobbied against the “Bill for the Protection of Passive Smokers’ Health” that was never approved.^12^ ^18^ Additionally, in 1996, he used his political contacts to help prevent the passage of a tobacco control bill in Chile.^19^ As of January 2009, he continues as President of Tabacalera de El Salvador^20^ and a FUSADES advisor.^21^

### International certification of FUSADES

In 1997, Zablah sought help from PM to obtain international certification for the air quality measurement capabilities of the FUSADES laboratory. Bruce Davies and Don Layden, Manager and Analytical Chemist respectively, from PM’s Scientific Affairs visited the laboratory. In a letter to Cesar Rodriguez, then Vice-President of PM Latin America, Zablah wrote:

I am very enthusiastic about their visit and especially with the possible project to assist the FUSADES Laboratory so that the air research center which FUSADES has can be certified on an international level and so that it can be *of use to Philip Morris for the measurements it has to make in the Central American area*. This would be a very important project for Philip Morris as well as for FUSADES.^22^ ^23^ [emphasis added]

Certification of the FUSADES laboratory also received support from top officials of PM Corporate Affairs Latin America. In a June 1997 letter to Cathy Ellis, PM Vice-President of World Scientific Affairs (WSA), Cathy L Leiber, Director of Corporate Affairs Latin America, wrote:

Both Jorge [Zablah] and I feel that meaningful, valid scientific studies in the areas of indoor and outdoor air quality conducted by a respected laboratory in Latin America, will be important to our *corporate goals* in the region.^24^ ^25^ [emphasis added]

To upgrade FUSADES, Davies hired LabStat, a Canadian-based laboratory. He worked with LabStat’s President and Laboratory Director, Bill Rickert^26^ to transfer testing protocols and methodologies to FUSADES. LabStat proposed to “… [Also] insure that FUSADES obtains national and internationally recognised accreditations (Conycet, ISO 25, ISO 37)”.^27^

LabStat planned to upgrade FUSADES in two phases. Phase one, costing $89 220, documented the state of preparedness of FUSADES to carry out SHS ambient air related analysis.^26^ Rickert billed PM an extra $9086.67 for a site review of the laboratory.^28^ ^29^ Phase two costs were $270 000 and included FUSADES personnel training and analysis of control samples under a new reorganisation scheme.^30^^–^32 A new analytical chemist, Ricardo Vives, was hired and assigned to air quality measurement issues that concerned PM.^33^ Vives travelled to Canada and received training in indoor air quality measurements at LabStat (Ricardo Vives, personal communication).

When asked about his collaboration with FUSADES, Rickert replied “Yes we were asked by PM USA to assess and provide advice to FUSADES on what was required for ISO 17025 Accreditation” (Bill Rickert, personal communication). However, when asked if LabStat was hired by PM in order that FUSADES could get accreditation, he replied “Actually the purpose was to audit the lab and tell FUSADES what, in our opinion had to be done BEFORE they could apply for accreditation” (William Rickert, personal communication).

An essential component of FUSADES upgrading was to obtain national and international accreditations.^27^ ^34^^–^36 The national CONACYT (Consejo Nacional de Ciencia y Tecnología or National Science and Technology Council) and International Organization for Standardization (ISO) certification processes, were part of the phase two LabStat upgrading plan.^35^ ^37^ FUSADES was rated qualified by LabStat for indoor air analysis by the end of 1998.^38^ CONACYT and ISO 17025 (formerly ISO 25) certifications were granted in 1999.^39^^–^43

Davies looked to outside laboratories for help^44^ with further upgrades, including Cigatam’s (PM affiliate in Mexico) cigarette testing laboratory,^45^ PerkinElmer’s branch in Costa Rica and LabStat in Canada.^31^ Additionally, Davies planned to ask PM for a $100 000 grant per year for 3 years for air quality measurements and laboratory personnel training.^46^

### The Latin America Scientific Network

The FUSADES laboratory initiative was part of the Latin America Scientific Network that included doctors and scientists as consultants under supervision of Davies.^47^^–^50 Of the 15 consultants, 9 were also part of the “Latin Project” established by PM and BAT in 1992.^6^ In a 1997 anonymous outline “A Strategic Framework: Toward Achieving Reasonable Smoking Policies”, FUSADES and the Network were to “*Contribute to the debate and correct misinformation pertaining to ETS* [environmental tobacco smoke] and related scientific issues” [emphasis added] and “Enhance capability to conduct regional IAQ [indoor air quality] analyses”[emphasis added].^51^

FUSADES would serve as an approved analytical laboratory where Network consultants would send their indoor/outdoor air samples for analysis.^36^ ^52^ However, other than the evidence from the Latin Project, we found no data suggesting that the Network was ever or is still active.

### Proposed funding options for FUSADES

FUSADES was the first step of PM’s effort to establish a network of ISO certified laboratories in Latin America. At the end of 1998 PM hired the services of HMS Group, a US limited liability company, to search for viable funding options and create a working plan for FUSADES that could be used as a model for other Latin American laboratories.^53^^–^55

Two funding options for FUSADES were proposed by HMS Group. First, an El Salvador-led effort in partnership with a public/private/university consortium. Second, a FUSADES-led effort (instead of a country-led effort) also in partnership with a public/private/university consortium.^56^ ^57^ HMS planned to contact the Ministry of Environment in El Salvador about establishing FUSADES as a test laboratory for El Salvador.^58^

Another possible source of funding for FUSADES was the humanitarian aid provided to El Salvador after hurricane Mitch.^59^ Donald Nelson, then Vice-President of Sales Development & Training for PM Latin America, emailed Davies “Given the attention that Central America is likely to get as a result of hurricane damage, this [FUSADES funding] may be something that could be attached to any bill involving aid to that area”.^60^ HMS proposed that FUSADES “should make an immediate all-out effort to obtain funding in support for its upgrade of laboratory facilities and development of a regional laboratory training centre as part of this reconstruction effort”.^61^

## DISCUSSION

Like its previous worldwide efforts,^2^ ^62^ ^63^ the tobacco industry established an air quality research centre in El Salvador to help achieve its corporate goals and support its commercial interests. PM provided financial and technical assistance to the FUSADES laboratory in El Salvador to turn it into a nationally and internationally certified air quality measurements centre. This laboratory would serve as a model to establish a laboratory network in Latin America.

**What this paper adds**

- The tobacco industry has created its own research institutions with the purpose of misleading the debate on second-hand smoke. By hiring scientists as consultants and using affiliated institutions, the industry has managed to divert attention from second-hand smoke as the major contributor to air quality deterioration to that of other air contaminants.
- Latin America did not have an air quality control laboratory that the industry could use to conduct its air quality research. By creating such a laboratory, the industry prepared to mislead public health efforts to regulate second-hand smoke.

Funding of FUSADES by the tobacco industry is a concern for two reasons. First, as part of El Salvador’s organised private sector, FUSADES has played an important role in the country’s political, economic and social life. Its recommendations are often taken into consideration by government authorities. Second, numerous analyses of pharmaceutical, nutrition, tobacco and chemical research show that industry-sponsored research results outcomes favour the sponsor’s product.^64^^–^67 Thus, FUSADES research would most likely favour the tobacco industry’s interests to divert the attention away from SHS as an air pollutant towards other outdoor air pollutants (eg, carbon monoxide).

A potential limitation of our study was the difficulty in obtaining the results of FUSADES air quality reports. We found two reports (1999 and 2005) prepared by SWISSCONTACT in Central America where FUSADES undertook air quality measurements in El Salvador.^17^ ^68^ Both focus on outdoor pollution, following the industry’s strategy of diverting the attention away from indoor air pollution where SHS is the major contributor. From 2005 until 2007 the country’s Ministry of Environment published yearly outdoor air quality reports undertaken by FUSADES.^69^^–^71 According to Regina Cortez, FUSADES Manager of the Environment Unit, since 2007 the Ministry would no longer use the laboratory and instead is collaborating with a local university (Regina Cortez, personal communication). According to Vives, air quality studies could no longer be performed once PM stopped supporting the laboratory in 1999. He assured us that FUSADES has never performed any indoor air quality studies but their intention to do so was the reason why PM collaborated with them (Ricardo Vives, personal communication). Both FUSADES employees were unable to provide any air quality studies or references beyond those that were found in the organisation’s website.

In summary, PM financial and technical assistance to the FUSADES laboratory in El Salvador sought to help its corporate goals as opposed to generate data to solve the problem of indoor and outdoor air pollution. Public health advocates and government officials should inquire about source of funding of indoor and outdoor air quality studies. In addition, the tobacco industry also funds non-governmental organisations to improve its corporate image.^72^
